# Supplementary material for: Simultaneous quantification of palbociclib, ribociclib and letrozole in human plasma by a new LC-MS/MS method for clinical application
Source: PLoS One. 2020 Feb 7;15(2):e0228822. doi: 10.1371/journal.pone.0228822 (PMC7006908; doi:10.1371/journal.pone.0228822)
Supplement: S1 Table — (DOCX) [file pone.0228822.s001.docx]

**S1 Table.** **Short term stability of PALBO, RIBO and LETRO.**

|  |  | **T = 4h (RT)** | | | **T = 72 h in autosampler (4°C)** | | |
| --- | --- | --- | --- | --- | --- | --- | --- |
| **Analytes** | **Nominal conc. (ng/mL)** | **Mean ± SD** | **Prec. %** | **Acc. %** | **Mean ± SD** | **Prec. %** | **Acc. %** |
| **PALBO** | 0.50 | 0.52±0.06 | 12.2 | 103.5 | 0.54±0.02 | 4.2 | 108.2 |
|  | 20.00 | 20.31±0.59 | 2.9 | 101.6 | 21.10±1.23 | 5.8 | 105.5 |
|  | 200.00 | 204.51±10.42 | 5.1 | 102.3 | 215.60±8.28 | 3.8 | 107.8 |
| **RIBO** | 20.00 | 18.86±0.74 | 3.9 | 94.3 | 21.24±1.45 | 6.8 | 106.2 |
|  | 800.00 | 751.88±27.56 | 3.7 | 94.0 | 815.31±36.72 | 4.5 | 101.9 |
|  | 8000.00 | 7218.92±402.94 | 5.6 | 90.2 | 8274.93±207.29 | 2.5 | 103.4 |
| **LETRO** | 1.00 | 0.88±0.06 | 7.1 | 88.2 | 0.94±0.07 | 7.8 | 93.9 |
|  | 40.00 | 37.95±1.78 | 4.7 | 94.9 | 44.45±0.19 | 0.4 | 111.1 |
|  | 400.00 | 372.54±24.58 | 6.6 | 93.1 | 426.23±4.43 | 1.0 | 106.6 |
