# Supplementary material for: Simultaneous quantification of palbociclib, ribociclib and letrozole in human plasma by a new LC-MS/MS method for clinical application
Source: PLoS One. 2020 Feb 7;15(2):e0228822. doi: 10.1371/journal.pone.0228822 (PMC7006908; doi:10.1371/journal.pone.0228822)
Supplement: S2 Table — (DOCX) [file pone.0228822.s002.docx]

**S2 Table. Stability after two freeze-thaw cycles.**

|  | **After 2 freeze-thaw cycles** | | | |
| --- | --- | --- | --- | --- |
| **Analytes** | **Mean ± SD** | **Prec. %** | **Acc. %** | **Mean ± SD** |
| **PALBO** | 0.56±0.08 | 14.2 | 111.9 | 0.56±0.08 |
| **RIBO** | 21.59±0.17 | 0.8 | 107.9 | 21.59±0.17 |
| **LETRO** | 207.66±9.03 | 4.3 | 103.8 | 207.66±9.03 |
