# Supplementary material for: Simultaneous quantification of palbociclib, ribociclib and letrozole in human plasma by a new LC-MS/MS method for clinical application
Source: PLoS One. 2020 Feb 7;15(2):e0228822. doi: 10.1371/journal.pone.0228822 (PMC7006908; doi:10.1371/journal.pone.0228822)
Supplement: S3 Table — (DOCX) [file pone.0228822.s003.docx]

**S3 Table.** **Long term stability (2 months) of PALBO, RIBO and LETRO: analytes stored in human plasma at -80°C and working solutions (methanol) stored at -20°C.**

|  |  | **Stored at -80ºC over 2 months (plasma)** | | | **Stored at -20ºC over 2 months (methanol)** | | |
| --- | --- | --- | --- | --- | --- | --- | --- |
| **Analytes** | **Nominal conc. (ng/mL)** | **Mean ± SD** | **Prec. %** | **Acc. %** | **Mean ± SD** | **Prec. %** | **Acc. %** |
| **PALBO** | 0.50 | 0.51±0.02 | 4.3 | 101.6 | 0.52±0.02 | 3.3 | 104.1 |
|  | 20.00 | 20.46±0.74 | 3.6 | 102.3 | 22.43±0.63 | 2.8 | 112.1 |
|  | 200.00 | 202.92±0.37 | 0.2 | 101.5 | 219.18±9.46 | 4.3 | 109.6 |
| **RIBO** | 20.00 | 21.10±1.05 | 5.0 | 105.5 | 19.52±0.30 | 1.5 | 97.6 |
|  | 800.00 | 789.37±28.89 | 3.7 | 98.7 | 885.44±35.25 | 4.0 | 110.7 |
|  | 8000.00 | 7699.21±117.73 | 1.5 | 96.2 | 8522.63±68.65 | 0.8 | 106.5 |
| **LETRO** | 1.00 | 0.88±0.01 | 1.4 | 88.3 | 1.05±0.03 | 2.7 | 105.1 |
|  | 40.00 | 39.58±0.90 | 2.3 | 99.0 | 44.12±1.25 | 2.8 | 110.3 |
|  | 400.00 | 386.66±6.61 | 1.7 | 96.7 | 413.47±6.07 | 1.5 | 103.4 |
